# Parent and child dietary changes in a 6-month mobile-delivered weight loss intervention with tailored messaging for parents

**DOI:** 10.3389/fpubh.2022.972109

**Published:** 2022-09-26

**Authors:** Brooke T. Nezami, Heather M. Wasser, Deborah F. Tate

**Affiliations:** ^1^Department of Nutrition, University of North Carolina at Chapel Hill, Chapel Hill, NC, United States; ^2^Department of Health Behavior, University of North Carolina at Chapel Hill, Chapel Hill, NC, United States; ^3^Lineberger Comprehensive Cancer Center, University of North Carolina at Chapel Hill, Chapel Hill, NC, United States

**Keywords:** intervention, parents, children, nutrition, text message

## Abstract

**Objective:**

To examine changes in parent and child dietary intake, associations between program adherence and parent dietary changes, and the association between parent and child dietary changes in a mobile-delivered weight loss intervention for parents with personalized messaging.

**Methods:**

Adults with overweight or obesity and who had a child aged 2–12 in the home were recruited for a randomized controlled trial comparing two types of dietary monitoring: calorie monitoring (Standard, *n* = 37) or “red” food monitoring (Simplified, *n* = 35). Parents received an intervention delivered *via* a smartphone application with lessons, text messages, and weekly personalized feedback, and self-monitoring of diet, activity, and weight. To measure associations between parent and child dietary changes, two 24-h recalls for parents and children at baseline and 6 months measured average daily calories, percent of calories from fat, vegetables, fruit, protein, dairy, whole grains, refined grains, added sugars, percent of calories from added sugars, and total Healthy Eating Index-2015 score.

**Results:**

Higher parent engagement was associated with lower parent percent of calories from fat, and greater days meeting the dietary goal was associated with lower parent daily calories and refined grains. Adjusting for child age, number of children in the home, parent baseline BMI, and treatment group, there were significant positive associations between parent and child daily calories, whole grains, and refined grains. Parent-child dietary associations were not moderated by treatment group.

**Conclusions:**

These results suggest that parent dietary changes in an adult weight loss program may indirectly influence child diet.

## Introduction

The rates of obesity in adults and children in the United States remain a public health concern, as 42% of adults had obesity and 19.3% of children aged 2–19 had obesity in 2013–2016 (1, 2). Obesity in childhood increases the risk of obesity and its comorbidities (e.g., heart disease, type 2 diabetes, and cancer) in adulthood (3–5), which highlights the need to improve dietary and activity behaviors starting at an early age. Targeting dietary intake in the home is a critical avenue for prevention, as children aged 4–13 are, on average, only meeting about 50% of the requirements for a healthy diet (6), and ~65% of the calories consumed by children are consumed in the home (7–9). Aspects of the home environment such as parent food intake, family meals, and availability of healthy foods are known to impact child dietary intake (10–20). Moreover, epidemiological research has consistently shown that children who have at least one parent who is overweight or obese are at high risk of gaining excess weight in childhood (21, 22). While the associations between parent and child weight and dietary intake have been well documented, less is known about how to target parent dietary behaviors and changes in the home environment to produce changes in child dietary behaviors.

There is some evidence showing that interventions that target parent weight loss can indirectly have a positive effect on child weight (23–27), which suggests that the mechanisms that improve parent weight status, such as changes in the home food and activity environment, also impact the child. There is also evidence that interventions targeting the entire family can have an impact on child dietary changes. For example, a study targeting an increase in family meals with parents and their children ages 8–12 found that children in the intervention group were less likely to consume SSB daily compared to the control group (28), and a food parenting intervention targeting low-income mothers as the agent of change for dietary intake in preschool-aged children found that children reduced their intake of energy from solid fats and added sugars (SoFAS) at 12 weeks (29). These two studies did not measure parent dietary intake, which precludes the ability to measure whether child dietary changes were associated with parent changes. Other family-based programs have measured both parent and child dietary intake and found parent-child associations in dietary changes, including fruit and vegetable intake among parents and their preschool-aged children (30), changes in high-calorie “red” foods and fruits and vegetables among parents and children ages 7-12 (31), consumption of grains among fathers and their children ages 5–12 (32), fruit, carbohydrates, and meals with vegetables among fathers and their children ages 5–12 (33), and energy intake from core (healthy) foods, nutrient-dense unhealthy foods, fast foods, breakfast cereals, and SSBs (34). All of these studies required intensive in-person contact with both the parents and children (30–34). Larger public health impact might be achieved if lower intensity programs that reduce parent and child contact time were readily available. However, little is known about whether parent dietary changes within a parent-only intervention have an impact on child dietary behaviors.

Two studies have exclusively targeted parents with overweight or obesity and measured both parent and child dietary outcomes. One study included a 3-month telephone coaching intervention for parents of children ages 2–10 (35) and did not produce changes in any parent or child dietary components. The other, a 6-month individual- and group-based counseling weight management program for parents of children age 7–18, measured fruit and vegetable intake at all time points, but resulted in no changes in parent or child intake (36). Given the importance of parent dietary behaviors and the home environment, it is critical to determine the most efficient and efficacious way to involve parents as the agent of change in promoting positive dietary changes in children.

The objective of this study was to examine data from a completed 6-month behavioral weight loss intervention for parents, the PATH (PArents Tracking for Health) study (37), that included personalized messaging *via* text and smartphone app to examine: (1) changes in parent and child dietary intake components from baseline to 6 months, (2) the associations between parent program adherence and parent dietary changes, and (3) the associations between parent and child dietary changes and if treatment group moderated any of these associations.

## Methods

### Study design and participants

The primary aim of the PATH randomized trial was to compare the efficacy of two smartphone-delivered behavioral interventions that differed in the approach to dietary self-monitoring, with either standard calorie monitoring (Standard) or simplified monitoring of high-calorie “red” foods (Simplified) (37). Given that parents are busy and may need simpler alternatives to weight loss that don't require detailed daily tracking of calories (38, 39), the Simplified group used a Traffic Light approach that categorizes foods as green, yellow, or red (40), and tracked only “red” foods (high-calorie foods such as sweetened beverages, desserts, processed salty snacks, fried foods, etc.). The Institutional Review Board at University of North Carolina approved the study. Recruitment of parent-child dyads occurred in 2019 primarily *via* email listservs and social media. Eligible individuals had a BMI between 25 and 50 kg/m^2^, were between the ages of 21 and 55, had at least one child in the home aged 2–12, were not currently pregnant or pregnant in the last 6 months, participated in <150 min of moderate-to-vigorous physical activity a week, and neither the adult or child participant had pre-existing medical condition(s) that preclude adherence to dietary changes or exercise. Parents completed informed consent for their own participation and their child's participation, and children aged 7–12 completed an assent form. Parent-child dyads (*N* = 72) were randomly assigned to the standard calorie monitoring group (Standard) or simplified monitoring group (Simplified).

#### Intervention elements in both groups

The intervention was based on Social Cognitive Theory and targeted constructs including self-regulation, self-efficacy, outcome expectations, perceived barriers, and observational learning (41). Parents were the primary target of the intervention. Children did not receive any direct intervention contact or guidance for activity and dietary changes. Hereafter, “participants” refers to parents, unless otherwise specified. Participants in both groups attended one in-person group session, followed by a remote program delivered *via* lessons and personalized automated weekly feedback in the PATH study smartphone app, plus 4–5 tailored text messages each week. Participants had three daily goals: (1) self-weigh on their smart scale, (2) wear their Fitbit activity tracker and meet a daily activity goal that gradually increased throughout the study as they met their goals, and (3) track their dietary intake and meet their daily goal. The 18 behavioral lessons addressed topics such as modeling healthy eating and exercise, setting limits, snacking and screen time, and parent-child communication. Lessons primarily addressed adult behavior change but were framed in the context of having children in the home, acknowledging that all members of the family unit can be barriers or facilitators to change, and that parent changes in healthy behaviors can have a positive impact on the child's behaviors. In addition, the app for both groups included a “Family Corner” section that advised on how to apply the information and strategies learned with their children in the home. Approximately 1 of the 5 text messages every other week focused on parenting skills that can promote positive and healthy behaviors in the home (Supplementary Table 1). The remaining text messages included alerts that new lessons and feedback were available in the app, motivational messages, and messages tailored to the parents' progress toward their dietary, activity, and self-weighing goals.

#### Standard group dietary self-monitoring

Participants in the Standard group received a calorie goal (1,200–1,800 kcal/day) and tracked their calories in the Fitbit smartphone app. Messages they received about dietary intake were specific to calorie tracking and their calorie goal.

#### Simplified group dietary self-monitoring

Participants in the Simplified group used the Traffic Light approach that categorizes foods as green, yellow, or red. They received a red food limit of 3–5 per day and tracked only “red” foods (high-calorie foods such as sweetened beverages, desserts, processed salty snacks, fried foods, etc.) in a Food Log within the PATH study app. Only participants in the Simplified group had access to this Food Log. Messages they received about dietary intake were adapted directly from the calorie messages to be specific to red food tracking and their red food limit.

### Measures

#### Dietary intake

Dietary intake was assessed using 24-h dietary recalls with blinded, trained dietary assessment staff *via* telephone at baseline and 6 months. Participants completed two telephone 24-h dietary recalls per parent and child at each time point (two parent dietary recalls and two parent-reported child dietary recalls). Staff were instructed to conduct the parent and child dietary recalls on the same day, when possible. Dietary recall information was entered directly into the Nutrition Data System for Research (NDSR), which was used to calculate average daily intake of the following dietary components for both the parent and child: total caloric intake (total kcal/day), percent of intake from fat (pct fat/day), total vegetables in cups (total veg/day), total fruit in cups (total fruit/day), protein in ounces (protein/day), dairy in cups (dairy/day), whole grains in ounces (whole grain/day), refined grains in ounces (refined grains/day), added sugars in grams (added sugars/day), percent of intake from added sugars (pct added sugar/day), and the Healthy Eating Index 2015 total score, a measure of diet quality based on the Dietary Guidelines for Americans (ranges from 0 to 100, with higher scores indicating better diet quality) (42).

#### Anthropometrics

Weight and height of parents were objectively measured by trained staff blinded to treatment assignment following a standardized protocol. Measurements were taken twice (three times when not within 0.1 kg and 0.1 cm for weight and height, respectively) and averaged. Weight and height was used to calculate baseline body mass index (BMI; kg/m^2^). Parents completed their child's weight and height assessments at their home. The child stepped on the parent's smart scale two times in a row, and the parent used a CDC standardized protocol to measure the child's height in centimeters two times in a row (43), then entered the child's weights and heights into an online form. The child's age, sex, and at-home weight and height measurements were used to calculate BMI z-scores based on the Centers for Disease Control and Prevention growth charts (44).

#### Program adherence and engagement

Dietary self-monitoring data (Fitbit food logs for the Standard group and PATH app Food Log data for the Simplified group) was the primary measure of program adherence and was used to calculate the average number of days per week that participants met their dietary goal (i.e., tracked their dietary intake and stayed at or below their calorie goal or red food limit; range 0–7). Program engagement was operationalized as number of total days that the PATH app was opened (range 0–184).

### Statistical analyses

Descriptive statistics were calculated for demographic variables and dietary component variables at baseline and 6 months for parents and children. Demographic variables were tested for their association with 6-month dietary outcomes using ANOVA for continuous variables and chi-square tests for categorical variables, and significant confounders were included as covariates in all analyses. Paired *t*-tests were used to test for changes over time in parent and child dietary component variables. To examine the association between parent adherence to the program and parent dietary changes, separate models regressed the 6-month value of the parent dietary component on (1) average number of days/week meeting the dietary goal, and (2) total days of app usage, controlling for baseline value of the dietary component, baseline BMI, number of children in the home, and treatment group. To determine the unadjusted association between change in parent dietary components and child dietary components from baseline to 6 months, linear regression was used to regress the 6-month value of the child dietary component on the parent 6-month value of the same dietary component, controlling for the child and parent's baseline values of that dietary component. An adjusted model controlled for child age in months, parent baseline BMI, number of children in the home, and treatment group. To determine if the parent-child dietary associations varied by treatment group, an additional model included all prior covariates plus an interaction term for treatment group by parent change in the dietary component.

## Results

The baseline characteristics of the original study sample are presented in Table 1. Parents were, on average, 40.0 years old (SD = 4.6), with a baseline BMI of 34.2 (SD = 6.4), and 94% female. Index children were an average of 6.4 years old (SD = 2.9), with an average BMI z-score of 0.47 (SD = 1.37), and 58% were female. Parent baseline BMI, child age, and number of children in the home were associated with changes in dietary component variables and were included as covariates in the analyses. All participants completed parent and child dietary recalls at baseline. At 6 months, 66 participants (92%) completed parent dietary recalls and 66 (92%) completed child dietary recalls, with no difference by treatment group (*p* = 0.68).

**Table 1:** Baseline characteristics by treatment group.

**Characteristic**	**Standard**	**Simplified**	**All participants**
	**(*n* = 37)**	**(*n* = 35)**	**(*N* = 72)**
	**Mean ±SD or *n* (%)**	**Mean ±SD or *n* (%)**	**Mean ±SD or *n* (%)**
Age	39.8 ± 4.7	40.2 ± 4.7	40.0 ± 4.6
Female	35 (94.6)	33 (94.3)	68 (94.4)
**Ethnicity**			
Hispanic/Latino	0 (0.0)	2 (5.7)	2 (2.8)
Non-Hispanic/Latino	37 (100.0)	33 (94.3)	70 (97.2)
**Race**			
Asian	0 (0.0)	1 (2.9)	1 (1.4)
Black or African American	5 (13.5)	4 (11.4)	9 (12.5)
Hispanic, Latino, or Cape Verdean	3 (8.1)	0 (0.0)	3 (4.2)
White	29 (78.4)	28 (80.0)	57 (79.2)
Other^a^	0 (0.0)	2 (5.7)	2 (2.8)
**Education**			
High school, vocational training, or some college	1 (2.7)	4 (11.4)	5 (6.9)
Bachelor's degree	17 (46.0)	10 (28.6)	27 (37.5)
Graduate or professional degree	19 (51.4)	21 (60.0)	40 (55.6)
**Marital status**			
Married or living with partner	35 (94.6)	29 (82.9)	64 (88.9)
Not married or living with partner	2 (5.4)	6 (17.1)	8 (11.1)
Weight (kg)	99.1 ± 21.6	91.0 ± 15.9	95.2 ± 19.3
BMI (kg/m^2^)	35.3 ± 6.8	33.07 ± 5.7	34.2 ± 6.4
Mean number of children in home	1.9 ± 0.7	2.0 ± 0.9	2.0 ± 0.8
Child age (years)	6.0 ± 6.8	6.8 ± 2.6	6.4 ± 2.9
Child female	21 (56.8)	21 (60.0)	42 (58.3)
Child in school or full-day childcare	33 (89.2)	30 (85.7)	63 (87.5)
Child weight (kg)^b^	24.3 ± 10.0	28.3 ± 14.0	26.3 ± 12.2
Child BMI z-score^b^	0.47 ± 1.42	0.47 ± 1.35	0.47 ± 1.37

a Other = checked response option “Other” and race is unknown.

b Out of n = 34 available child measurements in Standard and n = 34 in Simplified.

### Changes in parent and child dietary components

Means and standard deviations for baseline and 6-month values and means and confidence intervals for change values for all dietary components are reported in Table 2. There was a significant reduction in parents' total kcal (-271.6 kcal/day; 95% CI: −457.2, −86.1; *p* < 0.01), added sugars (−16.2 g/day; 95% CI: −27.0, −5.3; *p* < 0.01), and percent of kcal from added sugars (−2.24%; 95% CI: −4.05, −0.44; *p* < 0.05) from baseline to 6 months. There were no significant changes in any child dietary variables from baseline to 6 months.

**Table 2:** Means and standard deviations of dietary component variables for *n* = 66 parents and children with dietary data at both time points.

	**Baseline**	**6 Months**	**Change**
	**Mean (SD)**	**Mean (SD)**	**Mean (95% CI)**
**Average kcal/day**			
Parent	1,743.2 (594.2)	1,471.6 (543.2)	−271.6 (−457.2, −86.1)^**^
Child	1,451.6 (353.0)	1,507.6 (463.7)	56.0 (−68.2, 180.3)
**Average pct fat/day (%)**			
Parent	37.57 (8.73)	37.24 (9.57)	−0.33 (−3.20, 2.54)
Child	32.71 (6.50)	33.23 (6.62)	0.52 (−1.44, 2.48)
**Average total veg/day (cup)**			
Parent	1.59 (1.18)	1.57 (1.05)	−0.02 (−0.34, 0.30)
Child	0.60 (0.67)	0.72 (0.77)	0.12 (−0.10, 0.33)
**Average total fruit/day (cup)**			
Parent	0.59 (0.89)	0.55 (0.65)	−0.05 (−0.28, 0.18)
Child	1.07 (0.72)	1.06 (0.83)	−0.01 (−0.24, 0.22)
**Average whole grains/day (oz)**			
Parent	1.67 (1.94)	1.39 (1.51)	−0.28 (−0.82, 0.26)
Child	1.28 (1.48)	1.40 (1.21)	0.12 (−0.27, 0.51)
**Average dairy/day (cup)**			
Parent	1.32 (0.99)	1.03 (0.89)	−0.30 (−0.60, 0.01)
Child	1.98 (1.15)	1.92 (1.31)	−0.06 (−0.40, 0.29)
**Average total protein/day (oz)**			
Parent	5.62 (2.98)	5.45 (3.14)	−0.17 (−1.20, 0.86)
Child	3.26 (1.76)	3.67 (1.91)	0.41 (−0.20, 1.01)
**Average refined grain/day (oz)**			
Parent	4.34 (2.76)	3.69 (2.99)	−0.65 (−1.60, 0.31)
Child	4.83 (2.60)	4.86 (3.01)	0.03 (−0.87, 0.93)
**Average added sugar/day (g)**			
Parent	46.9 (38.3)	30.7 (28.4)	−16.2 (−27.0, −5.3)^**^
Child	37.4 (24.7)	36.1 (21.1)	−1.3 (−7.5, 4.9)
**Average pct kcal added sugars/day (%)**			
Parent	10.10 (6.43)	7.85 (5.42)	−2.24 (−4.05, −0.44)^*^
Child	9.92 (5.65)	9.72 (4.87)	−0.20 (−1.61, 1.21)
**Average HEI total score**			
Parent	54.85 (12.72)	55.74 (13.01)	0.90 (−3.18, 4.98)
Child	56.88 (11.83)	58.31 (12.09)	1.43 (−1.76, 4.64)

* Paired t-test p < 0.05.

** Paired t-test p < 0.01.

### Association between parent adherence and engagement and dietary changes

Average number of days a week meeting the dietary goal was negatively associated with parent change in total kcal, such that each additional day of meeting a dietary goal per week was associated with a reduction of 89 kcal (*p* < 0.05; Table 3). In addition, each additional day of meeting a dietary goal per week was associated with a reduction of 0.43 ounces of refined grains (*p* < 0.05). Total days of app usage was negatively associated with percent of fat from calories, such that each additional day using the app was associated with a 0.06% reduction (*p* = 0.05).

**Table 3:** Associations between parent dietary adherence and program engagement and dietary component changes.

	**Parent diet change and**		**Parent diet change and total**	
	**days/week met dietary goal** ^**a**^		**days of app usage** ^**a**^	
	***B***	***p***	***B***	***p***
Average kcal/day	−89.02	0.02	−2.75	0.12
Average pct fat/day (%)	−1.04	0.12	−0.06	0.05
Average total veg/day (cup)	0.05	0.45	0.004	0.16
Average total fruit/day (cup)	0.05	0.27	0.003	0.22
Average total protein/day (oz)	−0.29	0.21	−0.010	0.33
Average dairy/day (cup)	−0.09	0.16	−0.002	0.53
Average whole grains/day (oz)	−0.08	0.46	0.003	0.50
Average refined grains/day (oz)	−0.43	0.04	−0.013	0.20
Average added sugar/day (g)	−1.21	0.54	−0.041	0.65
Average pct kcal from added sugars/day (%)	0.06	0.88	0.002	0.90
Average HEI total score	1.36	0.13	0.07	0.10

a Regression of parent 6-month dietary component on total days of app usage or average days met dietary goal, controlling for number of children in the home, parent baseline BMI, and treatment group.

### Association between parent and child dietary changes

Decreases in parent total kcal were significantly associated with decreases in child total kcal in both unadjusted and adjusted models (*p'*s < 0.05; Table 4). Despite minimal changes, on average, in parent and child vegetables and whole grains, there was a positive parent-child association for both vegetables and whole grains (*p'*s < 0.05), though the association for vegetables was attenuated to non-significance in the adjusted model (*p* = 0.06). Each additional 1 cup of vegetables among parents was associated with an increase of ~0.2 cups of vegetables in children, and each additional ounce of parent whole grains was associated with an increase of 0.2 ounces of whole grains in children. In addition, there was a significant association between change in parent and child refined grains in unadjusted and adjusted models (*p'*s < 0.01), such that a decrease of one ounce of parent refined grains was associated with a decrease of 0.40 ounces of refined grains in children. No parent-child dietary associations varied by treatment group.

**Table 4:** Association between parent (IV) and child dietary component (DV) changes from baseline to 6 months and interaction by treatment group.

	**Unadjusted model** ^**a**^		**Adjusted model** ^**b**^		**Interaction of parent diet by**	
					**treatment group** ^**c**^	
**Child dietary component**	***B* (SE)**	**95% CI**	***B* (SE)**	**95% CI**	***B***	***p***
Average kcal/day	0.21 (0.10)^*^	0.005, 0.414	0.26 (0.11)^*^	0.048, 0.469	0.12	0.58
Average pct fat/day (%)	0.07 (0.09)	−0.102, 0.240	0.07 (0.09)	−0.111, 0.258	−0.24	0.22
Average total veg/day (cup)	0.20 (0.09)^*^	0.021, 0.374	0.18 (0.10)	−0.013, 0.367	−0.30	0.13
Average total fruit/day (cup)	0.18 (0.16)	−0.139, 0.507	0.10 (0.17)	−0.247, 0.450	−0.17	0.63
Average total protein/day (oz)	−0.09 (0.08)	−0.237, 0.065	−0.06 (0.08)	−0.219, 0.089	0.02	0.92
Average dairy/day (cup)	0.22 (0.17)	−0.127, 0.571	0.25 (0.18)	−0.110, 0.611	−0.11	0.75
Average whole grains/day (oz)	0.20 (0.10)^*^	0.004, 0.400	0.21 (0.10)^*^	0.015, 0.411	0.09	0.65
Average refined grains/day (oz)	0.39 (0.11)^**^	0.166, 0.623	0.41 (0.12)^***^	0.183, 0.646	0.11	0.63
Average added sugar/day (g)	0.15 (0.08)	−0.018, 0.322	0.16 (0.09)	−0.026, 0.342	−0.17	0.40
Average pct kcal from added sugars/day (%)	0.14 (0.11)	−0.075, 0.346	0.09 (0.11)	−0.132, 0.316	0.01	0.98
Average HEI total score	0.19 (0.11)	−0.031, 0.407	0.20 (0.11)	−0.033, 0.424	0.09	0.67

a Regression of child 6-month dietary component on parent 6-month dietary component, controlling for parent and child baseline values.

b Addition of covariates for child age, number of children in the home, parent baseline BMI, and treatment group.

c Addition of interaction term for treatment group by parent 6-month value of dietary component.

* Paired t-test p < 0.05.

*** Paired t-test p < 0.001.

## Discussion

This study demonstrated that parents participating in a smartphone-based behavioral weight loss intervention had positive changes in several aspects of their diet, including total kcal, added sugars, and percent of kcal from added sugars. Children, on average, did not have significant changes in any dietary components. Despite that, there were some positive associations between changes in parent dietary intake and child dietary intake. Thus, while mean scores for some dietary components did not reveal significant changes in the same direction across the full sample of parents and children, some changes that parents made were associated with similar changes in their children. Specifically, child changes in total kcal, whole grains, and refined grains mirrored the changes made by the parent. The parent-child association for vegetable intake was significant in the unadjusted model but not after adjusting for several covariates. These are similar to the dietary components that have shown prior parent-child associations over time in consumption of grains, carbohydrates, and vegetables (30, 32, 33). In this study there was no parent-child association for dietary components such as protein, dairy, and fat, which is also commensurate with prior findings (33, 45).

Importantly, total kcal and HEI score represent overall changes in dietary intake, whereas the remaining variables represent changes in specific dietary components. This sample of children who, on average, do not have overweight or obesity, would be expected to have increases in caloric intake as they grow. The significant mean reduction in parent kcal over time in conjunction with the significant parent-child association in total kcal changes suggests that the children who had the lowest increases in kcal were those whose parents had greater decreases in kcal. Given that both the calorie and red food approaches were designed to target a reduction in caloric intake to achieve weight loss, these findings suggest that the dietary changes made by the parents to reduce their overall caloric intake impacted the overall dietary intake of their children, as well. This is commensurate with a prior study showing similarities in reductions in energy-dense foods among both parents and children (34). Interestingly, the diet quality as measured by the HEI score did not change in either parents or children in this sample. This suggests that the dietary changes parents made to lose weight may have included small changes across various dietary components, and that these changes were highly variable across parents (e.g., some parents may have chosen to eat more vegetables and less protein, whereas others may have chosen to eat more protein, less dairy, and make no changes in their vegetable intake). Children appeared to have improvements in the total HEI-2015 score, though this did not reach significance. The average HEI score at 6 months was 58.3, which is slightly higher than the national average HEI score of 54.5 for children 2–5 and 53.8 for children ages 6–11 (46), but well under the guidelines for a healthy diet.

Prior studies that have found associations between aspects of parent and child dietary intake have included intervention components specifically targeting the child, such as character-based intervention content and positive reinforcement using rewards (30–32). This is one of the first intervention studies to demonstrate that solely targeting parent dietary changes can also produce changes in child diet when parents successfully make changes in their own diet. Similarly, the parent-child associations did not differ by treatment group. The Standard group used detailed calorie tracking with few guidelines other than a calorie goal, while the Simplified group tracked only red foods and limited their high-calorie foods to 3–5 per day. This difference in type of dietary changes made and method of tracking did not have an impact on the dietary components that were similar among parent-child dyads, which suggests that parental improvements in diet, regardless of whether they focus on reducing total calories or just high-calorie red foods, have the potential to improve child dietary intake.

One of the aims of the present analysis was to understand more about how parent engagement and adherence in the program influenced parent diet, and subsequently child diet. This study was not powered to detect mediation effects, thus the analyses examined if parent engagement was associated with parent dietary changes. The finding of a significant association between average days per week meeting the daily dietary goal and total kcal is consistent with the program's goals and highlights the importance of self-monitoring daily dietary intake and meeting the calorie goal (or red food goal). It is possible that parent-targeted interventions can indirectly influence child dietary patterns through parents' own adherence to dietary self-monitoring, likely *via* changes in the home environment and meals prepared in the home, though this was not measured in this study. It is unclear why total days of engagement with the study app was associated with parent changes in percent of intake from fat and no other dietary components. Given that total kcal and total fat intake are sensitive to social desirability bias in dietary recalls, but that is less true for percent of fat from kcal (47), it is possible that percent fat as measured at baseline was higher and more accurately reported than other dietary variables, and thus appeared to have a greater reduction during the intervention.

A limitation to this study is its small sample size and short duration of 6 months, which limits the ability to detect long-term parent-child associations in dietary changes, and, given the large range in child age, to test differences by developmental stage of the child. Similarly, both the parent and child dietary outcomes were measured at 6 months, which precludes a conclusion that the changes the parents made in their eating behaviors had a prospective effect on changes in the children's eating behaviors. However, given that the intervention content was directly targeted to the parent, including daily dietary goals, the recommendation to self-monitor intake daily, and text messages and weekly feedback reinforcing the parent's dietary progress, it is not likely that parent-child dietary associations would occur in the other direction (i.e., child dietary changes occurred first and would subsequently impact parent dietary changes). An additional limitation is that this study was not designed to determine the mechanism of parent-child dietary changes. Based on prior research, the most likely mechanism is the changes that were made in the home environment (18, 20). Parents in both treatment groups had dietary goals designed to help them lose weight and likely made many changes in the food and beverages available in the home, which could impact the meals prepared and the food consumed by the child when they are in the home.

Overall, this study found several modest associations between program engagement and parent dietary changes, as well as some associations between parent and child dietary changes. These preliminary findings suggest the ability to improve child dietary behaviors without directly including them in an intervention or program, and the role of parents as role models in the home when focusing on their own health and wellness goals. Larger randomized trials are warranted that specifically test the effect of low-intensity, parent-targeted programs for promoting improvements in child dietary behaviors.

## Data availability statement

The data analyzed in this study is subject to the following licenses/restrictions: The authors do not have permission to share raw study data due to requirements to protect the privacy of participants, in accordance with their informed consent (University of North Carolina at Chapel Hill IRB Study #17-3027). However, de-identified data related to this analysis may be made available upon reasonable request within 5 years of this publication. Requests to access these datasets should be directed to BN, bnezami@unc.edu.

## Ethics statement

The studies involving human participants were reviewed and approved by the Institutional Review Board, University of North Carolina at Chapel Hill. Written informed consent to participate in this study was provided by the participants' legal guardian/next of kin.

## Author contributions

BN and DT designed, implemented, and evaluated the original randomized trial. BN conceived the research question, conducted data analyses, and wrote the original draft. HW and DT provided feedback on the research question and analyses. All authors were involved in writing the paper and had final approval of the submitted and published versions.

## Funding

This project was supported by funding from the Karen Miller-Kovach Research Grant, provided by WW and The Obesity Society. This work was supported in part by the UNC Connected Health Applications and Interventions Core through a grant from the National Institutes of Health, National Institute of Diabetes and Digestive and Kidney Diseases (P30DK056350 to the UNC Nutrition Obesity Research Center) and/or from the National Institutes of Health, National Cancer Institute (P30CA016086 to the UNC Lineberger Comprehensive Cancer Center). The use of REDCap was supported by a grant from the Clinical and Translational Science Award Program of the Division of Research Resources at NIH (UL1TR002489).

## Conflict of interest

Author DT is a member of the Scientific Advisory Board for WW International and the Scientific Advisory Board for Wondr Health. The remaining authors declare that the research was conducted in the absence of any commercial or financial relationships that could be construed as a potential conflict of interest.

## Publisher's note

All claims expressed in this article are solely those of the authors and do not necessarily represent those of their affiliated organizations, or those of the publisher, the editors and the reviewers. Any product that may be evaluated in this article, or claim that may be made by its manufacturer, is not guaranteed or endorsed by the publisher.
