# Supplementary material for: Parent and child dietary changes in a 6-month mobile-delivered weight loss intervention with tailored messaging for parents
Source: Front Public Health. 2022 Sep 26;10:972109. doi: 10.3389/fpubh.2022.972109 (PMC9548804; doi:10.3389/fpubh.2022.972109)
Supplement: Supplementary file 1 [file Table_1.docx]

Supplemental Table 1. Example text messages

| **Type of Message** | **Examples** |
| --- | --- |
| Goal progress feedback | Hey there, Julie! Finish your week strong - log your foods and beverages in the Fitbit app to stay on track.  Stepping on the scale is a simple habit that takes just about 5 seconds a day. Use the handy Reminders app on your phone to set an alert to weigh in the morning so you don't forget!  You've met your active minutes goal all week long. Nice job! Don't forget, your activity during the weekend is important too. Make sure you're wearing and syncing your Fitbit daily!  You still have 2 days left in the week. This means 2 full days to track your red foods, reach your goals, and make healthy decisions. You've got this! |
| Motivational | Can you find 10 minutes just for yourself today? Reflect on why you want to lose weight and the changes you are making for you and your child.  Everyday gives you a fresh start and another chance to meet your goals. Today is your day! |
| Parenting-based | It's important for both parents and children to eat breakfast! A missed morning meal can deprive adults and children from essential nutrients and the energy needed to power through the day. Focus on whole grains (with no added sugar), fruit, and even lean proteins if you want!  As you begin week 12, come up with a goal that you and your child (or children) can accomplish together. Maybe half of your active minutes will include them? Maybe you'll both try a new vegetable this week? Involving your child in your healthy decisions will empower them to lead healthier lives as they grow and develop  Children seem to be constantly full of energy. Channel some of that energy as you play with your kids. You could play tag, go for a walk, or even push them on the swings. Every little bit counts. Plus, they'll love it too! |
